# Machine Learning Algorithm to Predict Obstructive Coronary Artery Disease: Insights from the CorLipid Trial

**DOI:** 10.3390/metabo12090816

**Published:** 2022-08-30

**Authors:** Eleftherios Panteris, Olga Deda, Andreas S. Papazoglou, Efstratios Karagiannidis, Theodoros Liapikos, Olga Begou, Thomas Meikopoulos, Thomai Mouskeftara, Georgios Sofidis, Georgios Sianos, Georgios Theodoridis, Helen Gika

**Affiliations:** 1 Laboratory of Forensic Medicine and Toxicology, School of Medicine, Aristotle University of Thessaloniki, 54124 Thessaloniki, Greece; 2 Biomic_Auth, Bioanalysis and Omics Lab, Centre for Interdisciplinary Research of Aristotle University of Thessaloniki, 57001 Thermi, Greece; 3 First Department of Cardiology, AHEPA University Hospital, Aristotle University of Thessaloniki, St. Kiriakidi 1, 54636 Thessaloniki, Greece; 4 Laboratory of Analytical Chemistry, Department of Chemistry, Aristotle University of Thessaloniki, 54124 Thessaloniki, Greece

**Keywords:** metabolic markers, ceramides, acylcarnitines, lipids, biomarkers, coronary artery disease, SYNTAX score, atherosclerosis, acute coronary syndrome, metabolomics

## Abstract

Developing risk assessment tools for CAD prediction remains challenging nowadays. We developed an ML predictive algorithm based on metabolic and clinical data for determining the severity of CAD, as assessed via the SYNTAX score. Analytical methods were developed to determine serum blood levels of specific ceramides, acyl-carnitines, fatty acids, and proteins such as galectin-3, adiponectin, and APOB/APOA1 ratio. Patients were grouped into: obstructive CAD (SS > 0) and non-obstructive CAD (SS = 0). A risk prediction algorithm (boosted ensemble algorithm XGBoost) was developed by combining clinical characteristics with established and novel biomarkers to identify patients at high risk for complex CAD. The study population comprised 958 patients (CorLipid trial (NCT04580173)), with no prior CAD, who underwent coronary angiography. Of them, 533 (55.6%) suffered ACS, 170 (17.7%) presented with NSTEMI, 222 (23.2%) with STEMI, and 141 (14.7%) with unstable angina. Of the total sample, 681 (71%) had obstructive CAD. The algorithm dataset was 73 biochemical parameters and metabolic biomarkers as well as anthropometric and medical history variables. The performance of the XGBoost algorithm had an AUC value of 0.725 (95% CI: 0.691–0.759). Thus, a ML model incorporating clinical features in addition to certain metabolic features can estimate the pre-test likelihood of obstructive CAD.

## 1. Introduction

In an ever-changing environment with substantial medical achievements, coronary artery disease (CAD) remains the leading cause of mortality worldwide [1]. Therefore, current research predominantly focuses on the efficient prevention, risk-stratification, and management of patients with CAD to optimize their prognosis. Concurrently, several basic, translational and clinical research efforts aim to determine the etiological mechanisms underlying CAD pathogenesis and identify lifestyle-dependent metabolic risk factors or genetic and epigenetic parameters responsible for CAD occurrence and/or progression [2]. Thereby, clinicians could ultimately develop feasible and accurate risk assessment and prediction models with the potential to be incorporated into routine clinical practice.

Undoubtedly, as we have already entered the age of precision medicine, novel and promising CAD stratification strategies, based on the “-omics” fields, such as metabolomics, become even more salient [3,4]. Metabolic profiling based on sophisticated analyses can reveal serum metabolites whose levels could serve as a direct functional readout of the physiological state of an organism, thereby, reflecting the onset and progression of CAD [5]. Metabolic profiling data and publications on metabolic markers related to cardiovascular diseases have increased exponentially during the last decade, and some metabolites-based risk scores have been already developed; however, most investigations failed to translate into clinical benefit [6]. This might be associated with the large volume, challenging structure, and nonlinear interaction of metabolomics data, which render the conventional data analytic strategies less effective for such data characterization, annotation, and integration into risk scores [7]. Hence, the metabolomics community eagerly awaits to adopt novel mathematical and computational tools, able to refine data analysis and exploit the advanced applications of mass spectrometry to metabolic phenotyping [8].

To this end, machine learning (ML), a branch of artificial intelligence (AI), has been increasingly utilized across metabolomics studies due to the inherent nonlinear data representation and the ability to rapidly process large and heterogeneous data [7,9]. Although ML-based big data utilization is still in its infancy across cardiovascular medicine and still has some innate weaknesses (e.g., ‘black-box’ criticism, lack of design standardization, and limited applicability to clinical trials), ML techniques have been already applied to identify unknown CAD risk factors, automate imaging interpretation, enhance clinical decision-making, and bridge the gap between disease pathogenesis and phenotyping, facilitating precision medicine [10,11,12]. More accurate ML-based CAD prediction would empower clinicians with enhanced diagnosis, risk stratification, and ultimately, management of CAD patients, whilst potentially minimizing the necessary interventions [13,14]. Nevertheless, to the authors’ knowledge, there is not yet any clinically oriented ML-based approach incorporating metabolic markers analyses for the prediction of obstructive CAD among patients undergoing invasive coronary angiography (ICA).

Against this background, we sought to develop an accurate ML model, utilizing clinical and metabolite data from a real-world population undergoing ICA, to predict patients likely to have obstructive CAD on ICA and to assess its effectiveness in combination with an established clinical risk stratification algorithm. We hope that this pretest assessment tool could provide a framework that would guide the establishment of novel metabolic biomarkers for CAD development and would hopefully provide physicians with clinical decision support to optimize referrals to ICA versus noninvasive diagnostic modalities. 

## 2. Materials and Methods

### 2.1. Study Population and Eligibility Criteria

The CORLIPID trial (NCT04580173) is a non-interventional cohort trial, which enrolled 1065 adult patients without prior CAD undergoing ICA in AHEPA University Hospital of Thessaloniki within the period of July 2019–May 2021, and aimed to associate CAD severity with patients’ serum metabolic profile [15]. Prior percutaneous coronary intervention (PCI) or coronary artery bypass grafting (CABG), along with cardiopulmonary arrest at presentation or severe comorbidity with a life expectancy of less than 1 year constituted the exclusion criteria of the study.

### 2.2. Study Outcomes

The primary outcome of this study was to combine clinical characteristics with established and novel metabolic biomarkers aiming to develop an obstructive CAD risk prediction model based on an ML approach. The secondary study outcome was to distinguish patients with acute coronary syndrome (ACS) from those with chronic coronary syndrome (CCS) through metabolite pattern differentiation.

### 2.3. Metabolic Marker Analyses

Venous blood samples were collected prior to ICA execution. Mass spectrometry analytical methods were developed and applied to define serum levels of specific lipid biomarkers: four ceramides, 13 acyl-carnitines, and a comprehensive profile of 23 fatty acids. Galectin-3 was also determined for all study participants, while other protein levels, including adiponectin, apolipoproteins (A1 and B), and neutrophil gelatinase-associated lipocalin (NGAL) were measured for a subset of study participants (216, 405, and 119 patients, respectively). 

### 2.4. Angiographic Analyses

All coronary angiograms were visually assessed by two blinded experienced invasive cardiologists (EK and GS); each cardiologist calculated the SYNTAX score [16] for each patient and any disagreements were resolved through consensus. Patients were categorized into corresponding groups based on the indication for ICA [ACS, CCS] and on the severity of CAD using the SYNTAX score. In categorical terms, obstructive CAD was defined as ≥50% stenosis of any major epicardial vessel of >2 mm in diameter [17].

### 2.5. Statistical Considerations

Conventional statistical analysis of the data was performed through IBM SPSS Statistics for Windows, version 26 (IBM Corp., Armonk, NY, USA) and Microsoft Excel. Clinical, procedural, and demographic data are presented as the mean ± standard deviation (SD) or frequencies and percentages as appropriate. Our data were not parametric, thus, categorical differences between patient groups were evaluated by the χ^2^ test for discrete clinical variables, while differences in paired concentrations were evaluated by the Wilcoxon signed-rank test. To assess the differences in serum concentrations or measured areas among study groups, the Mann–Whitney U or Bonferroni corrected for multiple comparisons Kruskal–Wallis test was used. Statistical significance was defined as a value of *p* < 0.05.

### 2.6. Machine Learning Algorithm

Patients included in the analyses were characterized by a total of 8 readily available demographic and clinical variables, including age, gender, CAD risk factors (including diabetes mellitus, hypertension, dyslipidemia, smoking, family history of premature CAD, and body mass index), along with 12 biochemical and 52 novel protein-markers and metabolites variables available in our dataset. Within the selected variables, no further clinical metrics are included with the aim to establish an application feasible also in a non-hospital diagnostic setting.

In order to produce an efficient, reliable, and accurate SS prediction model, ML methods were applied, using XGBoost as the algorithm of choice. XGBoost is a non-linear, supervised algorithm, capable of handling both regression and classification prediction problems, which has recently been dominating applied ML competitions for structured and tabular data.

XGBoost (stands for eXtreme gradient boosting) belongs to the more general category of decision-tree-based ensemble ML algorithms which are considered among the best options for the analysis of small-to-medium structured data. In particular, XGBoost is an optimized gradient boosting algorithm, which in turn is an evolution of the family of boosting ensemble algorithms. Boosting algorithms build the sequential models in such a way as to minimize the errors of previous models and enhance the impact of high-performing models [18,19]. Gradient boosting is a special case of boosting which implements a gradient descent algorithm to minimize errors in sequential models [20]. Finally, XGBoost further improves gradient boosting using a combination of software and hardware optimization techniques, achieving superior results in terms of execution speed and model performance [21].

The aforementioned software and hardware optimization techniques include, among others, parallelization in the building of successive models, decision tree pruning to a specific depth, regularization [22] (both *l*1 and *l*2) to prevent overfitting, and sparsity awareness for the optimal handling of datasets with missing values, etc. The effect of these techniques is controlled by a series of hyperparameters of the XGBoost algorithm, which are set to their optimal value before the analysis of each dataset. Evaluation metrics equations are presented in the Supplementary Materials.

### 2.7. Prediction Model Evaluation

**Figure 1:**
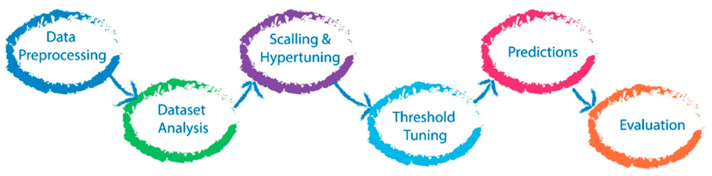
Data analysis workflow.

To evaluate the performance of the ML SS prediction model, the 10-fold cross-validation (10CV) technique was used, which is completed in 10 consecutive stages [23]. Initially, the samples (rows) of the dataset under study are randomly divided into 10 equal-sized segments. At each stage of the technique, a different segment is selected and used as the test set with which the performance of the algorithm is evaluated, while the remaining 9 segments form the training set with which the algorithm is trained. In this way, each segment of the dataset is used exactly once as a test set. At each stage and before training the algorithm, the processes of data scaling and hyperparameter tuning are implemented, which are described in the following subsections. By combining the predictions for the individual test sets, the predictions for the overall dataset are obtained, which are used for the final evaluation of the predictive algorithm using the appropriate evaluation metrics. Figure 1 illustrates the general methodology followed for the dataset analysis.

### 2.8. Post-Hoc Model Correction

Aiming to improve the predictive capability of the CorLipid algorithm, we combined post hoc the XGBoost model with the Diamond–Forrester score for CCS patients and with the Grace score for ACS patients [24,25]. Such a strategy has been applied in previous relevant studies, for example, in the study by Al’ Aref et al., (2020) [26], where an XGBoost algorithm was combined with the Diamond–Forrester score for 13,054 CCS patients from the international CONFIRM registry.

### 2.9. Data Scaling

Before their use and in order to be better exploited by the predictive algorithm, the values of each individual feature (column) of the dataset are appropriately scaled so that the resulting distribution exhibits a mean of 0 and a standard deviation of 1. This process is repeated at each individual stage of the central 10CV technique. The scaler used is first fitted on each individual training set and then applied to both the training and the corresponding test set.

### 2.10. Hyperparameter Tuning

As mentioned previously, the optimization techniques inherently used by the XGBoost algorithm are controlled by a set of hyperparameters. The hyperparameters are an important component of any ML algorithm playing a central role in determining the structure, complexity, and performance of the resulting predictive models [27]. In the present analysis, hyperparameter tuning is implemented in each individual stage of the central 10CV technique. A secondary 10CV procedure (nested CV) is applied to each individual training set in order to determine the optimal hyperparameter values for the specific part of the dataset. In each case, a total of 200 randomly selected hyperparameter sets of values are evaluated using Logloss (Equation (S5) Supplement) as the loss function. The overall best hyperparameter values set were then used for the fitting of the predictive model. Table S1 contains the hyperparameters optimized for the XGBoost algorithm, along with their respective ranges of investigated values.

### 2.11. Probability Threshold Tuning

The evaluation of the performance of a predictive binary classifier usually assumes a default probability threshold value of 0.50 in order to assign predicted probabilities to a given class. In order to reduce the proportion of false negative (FN) events, a separate analysis of the samples’ predicted probabilities is performed, where the proportion of FN events resulting in different values of the probability threshold is calculated. The value where at most 1% (or 5%) of samples belonging to the positive class are classified as FN is selected and used for the final evaluation of the predictive model. The analysis is carried out using in-house Python scripts.

### 2.12. Code Development

The programming part of the present analysis was implemented on a Linux-based desktop PC (Ubuntu 20.04.2 operating system, kernel v5.11.0, AMD Ryzen 5 3600 CPU, 64 GB RAM) using the JupyterLab web-based development environment. Code development was implemented using the Python (v3.8.10) programming language and the following main libraries: ipython v8.0.0, jupyterlab v3.2.8, matplotlib v3.5.2, numpy v1.22.4, pandas v1.4.2, scikit-learn v1.1.1, scikit-posthocs v0.6.7, scipy v1.8.1, seaborn v0.11.2, xgboost v1.6.1.

Code used in this project is available at the following repository: https://github.com/TheoLiapikos/Syntax_Score_prediction_model_for_CV_patients_using_XGBoost_Classifier (accessed on 27 July 2022).

## 3. Results

### 3.1. Baseline Characteristics

Our analysis includes data from 958 out of the 1065 study participants enrolled in the CorLipid trial, due to the unavailability of clinical and laboratory data for some of the samples. Almost 3 out of 4 study participants (73.4%) were of male gender. Moreover, 55.6% of our population presented with ACS, while the remaining patients underwent ICA due to CCS. Of the 533 patients suffering from ACS, 170 presented with NSTEMI, 222 with STEMI, and 141 with unstable angina (17.7%, 23.2%, and 14.7% of the total population). Median age of the total population was 65 years old (95% Cis: 64–66) and median SS was equal to 10 (95% Cis: 9–12). Two hundred and seventy-seven patients (28.9%) had non-obstructive CAD according to the coronary angiogram assessment, while 210 patients (21.9%) suffered from severe CAD (SS > 22). Almost half of our population (50.8%) were under statin medication. Baseline clinical and demographic characteristics are presented in Table 1 and Table 2.

### 3.2. Descriptive Analyses of Categorical and Continuous Variables According to CAD Subgroups

In our population, the male-to-female ratio was not different amongst the studied CAD subgroups (STEMI, NSTEMI, stable and unstable angina). The percentage of hypertensive and dyslipidemic patients differed across those groups (Table 3; *p* < 0.05). Family history of premature CAD was more evident in the STEMI subgroup compared to patients with stable angina (*p* = 0.012). 

The assessment of continuous variables based on CAD subgroups is illustrated in Table 4. Mean GRACE score and mean troponin, glucose, and SGPT values were significantly higher in patients with STEMI, compared to the rest subgroups (*p* <0.05).

Focusing now on the primary aim of the CORLIPID study, the comparison of metabolic biomarkers among the CAD subgroups yielded some significant differences as detailed in Table S4. 

Regarding ceramides, patients with stable angina had significantly lower measured C16:0 and C18:0 ceramide levels compared to patients with NSTEMI and STEMI. C24:0 and C24:1 were substantially higher in STEMI patients compared to patients with unstable and stable angina. Regarding acylcarnitines, five of those species showed significant level variations, with C5 carnitine having higher mean values in STEMI patients compared to patients with unstable angina, and C10, C16 C18.1, and C18.2 carnitines having lower mean values in STEMI compared to stable angina. Lipids showed also significant variation amongst CAD groups with most lipids being lower in the stable angina group than in ACS, except for C20:1n11 and C20:2 cis lipids which had lower values in STEMI compared to stable angina (Table S4). 

### 3.3. Metabolite Analyses According to SYNTAX Score Groups

In Supplementary Tables S2 and S3, we present the results from the descriptive analyses of categorical and continuous study variables, as well as the biochemical parameters according to CAD severity groups (SS subgroups: SS = 0, 1–22, >22). Mean GRACE score and mean troponin values were significantly higher in the high-severity group, while patients with diabetes mellitus (DM) and those presenting with higher glucose levels were at higher risk for severe CAD (*p* < 0.05).

The results deriving from the determined metabolites are presented in detail in Table S5, as compared among the SS groups. Regarding the protein markers evaluated, only ApoB/ApoA1 ratio differed significantly among the SS groups, with its lowest values being observed across the SS = 0 group. As for ceramides, C18:0 levels were significantly lower in the SS = 0 group compared to the other two groups. Mean values of the C4 and C5 acyl-carnitines were also significantly lower in the SS = 0 group, whereas C16 and C18:2 acyl-carnitines were significantly lower in the SS > 22 group. Regarding the fatty acids, mean C17:1 and cis C18:1 values were significantly lower in the SS = 0 group.

### 3.4. ML Results

**Figure 2:**
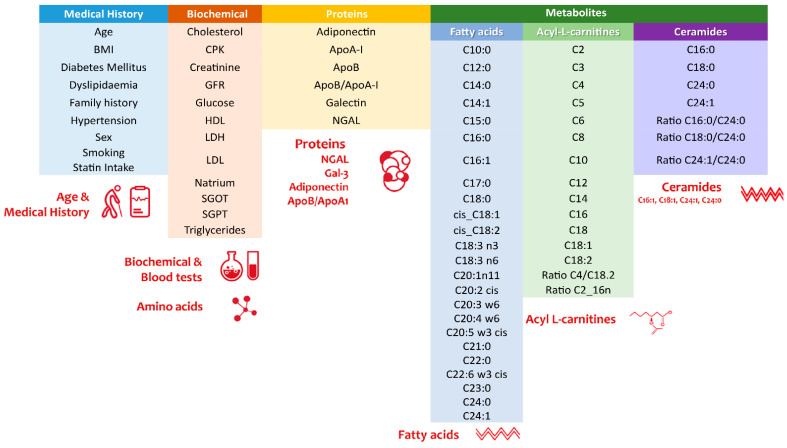
The 73 parameters that constitute the CorLipid algorithm input biomarker panel. ApoAI: apolipoprotein AI, ApoB: apolipoprotein B, NGAL: neutrophil gelatinase-associated lipocalin, Gal-3: galectin-3.

A total of 958 serum samples with 73 selected parameters were used as the algorithm dataset. The panel (see Figure 2) selection was based on available biochemical and metabolic markers and anthropometric and medical history variables that were recorded in the CorLipid dataset and presented herein. 

**Figure 3:**
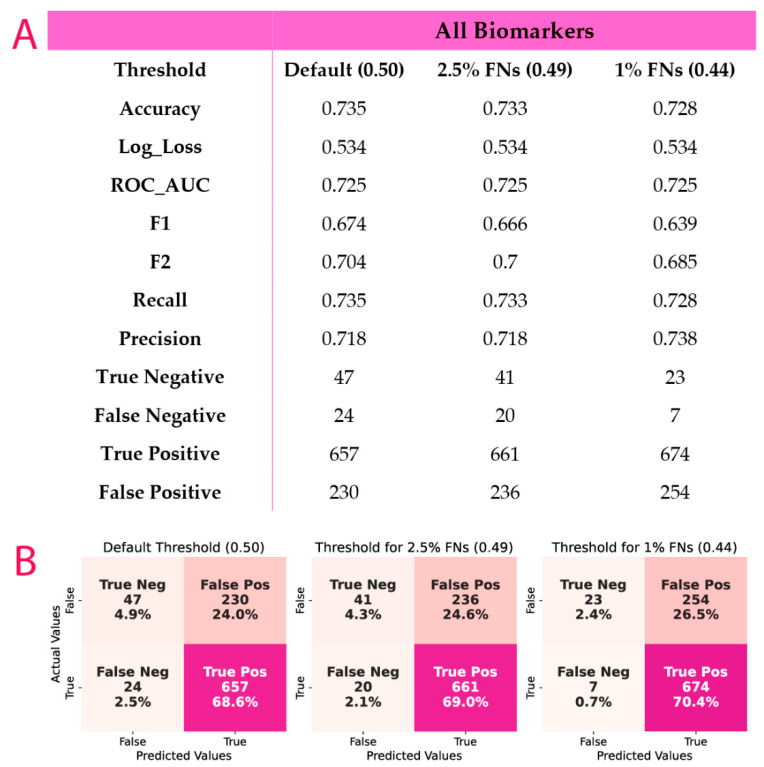
(**A**) Probability threshold and all quality metrics for the CorLipid algorithm. (**B**) Confusion matrices for true false positive and negative for the model with different false negative thresholds. FNs: false negative predictions, expressed as a percentage of the sum of FN and TP, FNs = FN/FN + TP).

All 73 parameters were used in the algorithm without any imputations or sample removal for empty cells thus leaving the dataset intact. The performance of the XGB algorithm on the full dataset to separate patients into: patients with SS = 0 and those with SS ≥ 1, was acceptable with an AUC value of 0.725 (95%Cis: 0.69–0.76). The evaluation of the performance of the developed model is presented in Figure 3. 

### 3.5. Post-Hoc Model Correction

**Figure 4:**
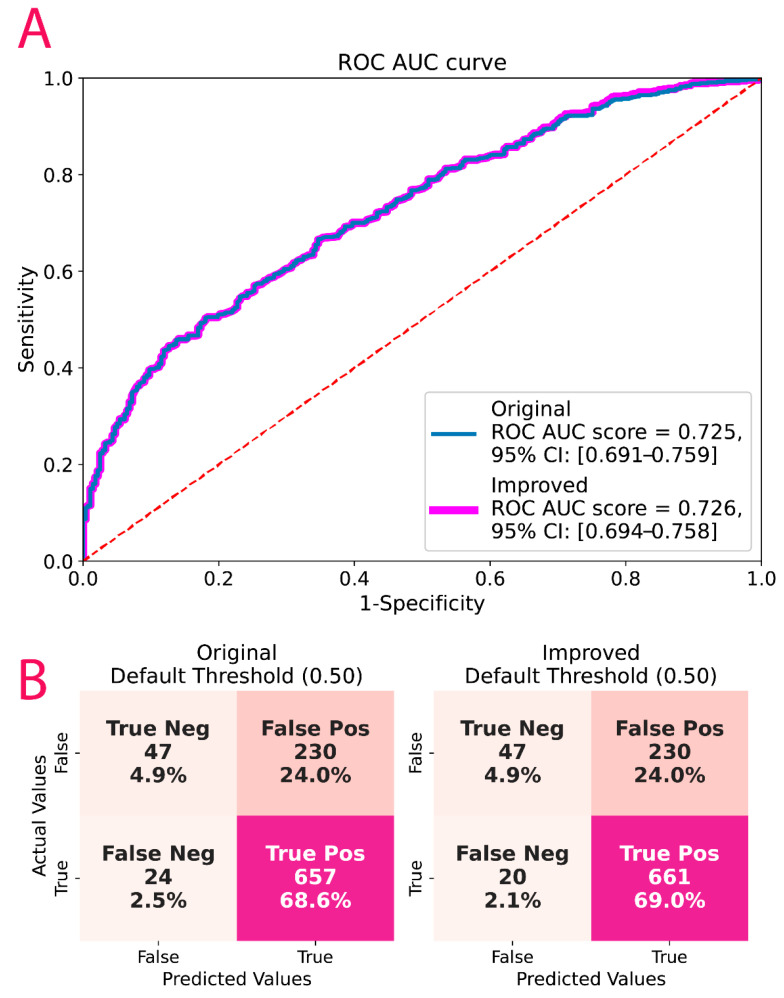
(**A**) Original and corrected ROC AUC of the CorLipid algorithm, (**B**) confusion matrices for true false positive and negative for the original and corrected models.

After combining XGBoost with Diamond–Forester and GRACE scores for CCS and ACS patients, respectively, there was no difference in algorithm performance, but the proportion of false negatives decreased with a small increase in false positives. Figure 4 includes the combined ROC AUC along with the FN percentages for both the original and the corrected models. 

## 4. Discussion

In this study, a number of specific lipid metabolites were determined by three targeted metabolomics methods to identify CAD-related serum metabolic biomarkers. We screened their potential as biomarkers serving for the non-invasive detection of obstructive CAD through a comprehensive XGBoost approach. The combination of the large input dataset containing several metabolic features with the ML methods constitutes the novelty of the presented study. This study is considered a preliminary approach; it is vital to further validate our results in larger datasets. Our results may be useful for utilizing metabolic data to improve early CAD prediction and may offer insights into the metabolic pathways involved in CAD pathogenesis. Furthermore, this clinical model will hopefully trigger further research efforts investigating whether a panel with some of those metabolites could enhance the diagnostic yield of ICA through optimized patient selection.

### 4.1. Metabolites in Cardiovascular Diseases

The field of cardiovascular metabolomics has seen substantial growth during the last decade. Most studies have been performed in less clinical settings aiming to gain deeper insight into pathophysiological interactions of metabolites and disease states [28,29]. A recent study briefly overviews the existing cardiovascular metabolomics studies, and makes clear that glucose, fatty-, and amino- acid metabolism perturbations are associated with the development of atherosclerosis and ischemic cardiomyopathy [6]. 

Targeted metabolomics have been already utilized for the discovery of CAD biomarkers with the aid of ML, revealing serum sphingolipids as cholesterol-independent biomarkers of CAD [30]. Based on targeted LC-MS/MS lipidomics, sphingolipid species were found to be positively associated with CAD. Other ML methods have also identified metabolic signatures that predict the risk of recurrent angina in patients discharged after PCI based on broad-spectrum LC-MS/MS targeted metabolomic data which were acquired by a method monitoring 606 MRM channels [31]. Atargeted SPE-LC-MS/MS method has been also applied for the analysis of omega-6-derived eicosanoids in the serum of CAD patients [32] to investigate their inflammatory response to CAD risk factors. Since alterations in xanthine oxidase activity are known to be pathologically associated with CAD, blood purine metabolite-based ML models have been developed for risk prediction, prognosis, and diagnosis of CAD [33]. The levels of xanthine and uric acid were proven to be critical in the development of ML models for primary/secondary prevention or diagnosis of CAD.

Several ceramides, phosphatidylcholines, and acylcarnitines have been recently linked with the incidence and progression of CAD. More specifically, in a multinational cohort “Biomarkers for Cardiovascular Risk Assessment in Europe” of more than 70,000 individuals, five phosphatidylcholines were significantly associated with increased risk of incident CAD and showed similar prognostic values as individual classic risk factors [34]. Moreover, our previous works based on the CorLipid dataset demonstrated that serum acylcarnitine levels are significantly associated with the SS, whilst the same applies to ceramide levels of diabetic individuals [35,36]. Elevated levels of specific serum ceramide species have been also linked with larger thrombus burden showing that ceramides emerge as potential mediators and prognostic biomarkers of CAD [37]. Furthermore, metabolic profiling technologies have been also utilized to reveal the prognostic course of CAD patients, either through a traditional risk score (e.g., CERT2 score) or through an ML algorithm (e.g., random forest algorithm) [38,39,40]. 

Thus, it is evident that as sample sizes [8] and the number of measured metabolites progressively increase in epidemiological settings, the conjunction of metabolites data across studies with other clinical and biochemical data will bolster our understanding of the cardio-metabolic background of CAD. Metabolic phenotyping paves the way to new mechanistic understanding and therapies, as well as improves the risk prediction of CAD patients. 

To that end, non-linear ML approaches for metabolite data seem to be very promising due to their non-linear nature and the existing interactions between multiple metabolite predictors and endpoints [28]. Nevertheless, selecting the optimal ML model for a given dataset is quite challenging since the choice depends on data properties and the project goal [41]. The implemented frameworks in such studies include random forest, deep learning and extreme gradient boosting (XGBoost) approaches that aimed to capture the metabolic complexity of several diseases [28,42]. The predictive capability of the XGBoost algorithm for the stratification of metabolic phenotypes seems to outperform other classification ML algorithms. 

However, an acceptable AUC cut-off to be used in clinical practice and the appropriate algorithms to be applied in metabolite datasets remain to be assessed, since the application of ML concepts is substantially limited by the unavailability of appropriate clinical datasets. An ML model that incorporates clinical features could lead to better risk stratification and help guiding subsequent management. An example of such a model has been previously communicated by Al’ Aref et al. [26], where a combination of XGBoost with the Diamond–Forrester score for 13,054 CCS patients of the international CONFIRM study was applied. Therefore, a post hoc correction of the CorLipid algorithm was performed in combination with Diamond–Forester and GRACE risk-stratification scores for CCS and ACS patients, respectively, and there was a decrease in the FN percentage; however, there was no significant increase in the generated AUC ROC. Hence, the post hoc corrected model might be more suitable for clinical use and not for the general public as the original CorLipid model, since it warrants an improvement in its predictive capability in conjunction with clinically available scores.

### 4.2. Coronary Artery Disease Prediction

From the point of statistical modeling, the prediction of CAD is a widely studied problem either through traditional (one-dimensional) regression analyses or through ML algorithms. The target of ML approaches is to specifically interpret how risk factors affect the outcome [43]. According to a recent meta-analysis on 45 cohorts encompassing a total of 116,227 individuals and using ML (CNN, SVM, RF, custom-built and boosting algorithms) for the prediction of CAD, the prediction of CAD with boosting algorithms was associated with pooled AUC of 0.88 (95% CI 0.84–0.91), sensitivity of 0. 86 (95% CI 0.77–0.92), and specificity of 0.70 (95% CI 0.51–0.84) [44]. The ensemble methods (such as the one implemented herein, XGBoost) use the boosting procedure to combine stumps of trees. This can be loosely conceptualized as forming an overall prediction by aggregating the predictions of many simpler predictive models. This might seem similar to the process of deriving a clinical diagnosis for a patient by utilizing consultations from many specialists, each of whom would look at the patient in a slightly different way. 

There is an anticipation that AI will result in a paradigm shift toward precision cardiovascular medicine in the near future [45]. Novel research strategies exploiting the ML powers could help clinicians in the prediction of patients that would benefit from invasive or non-invasive diagnostic modalities [46]. ICA constitutes the gold-standard test for CAD diagnosis; however, better pretest assessment could ultimately improve patient safety and decrease healthcare costs by optimizing referral for outpatient ICA [47]. 

### 4.3. Limitations, Strengths and Further Research

When interpreting our outcomes, some caveats could be recognized. The sample size could be considered relatively limited, as compared to other ML studies on CAD prediction, whilst the general lack of training and validation data limit the generalizability of our findings. Therefore, a more detailed input space and a larger external dataset of patients may ensure the applicability of our model as an effective multimodal prediction scheme. The practical applicability of this algorithm might also be somewhat restricted due to the requirement of expensive instrumentation and trained personnel for data extraction and interpretation. 

Nevertheless, the present study included the largest dataset of metabolites analyzed using targeted methods for ceramides [48], acylcarnitines [49] and fatty acids [50], to date, used for the development of a predictive ML score for the presence of obstructive CAD, as assessed through the SS. The created model is unique for several reasons. First, this ML-based predictive model was generated based on a diverse real-world cohort and did not require the execution of specialized clinical procedures, such as echocardiography or other imaging assessment tests. The developed algorithm solely requires patients’ serum extraction and the documentation of baseline medical history and demographic parameters. Implementing this metabolites-based model as part of a point-of-care decision could be particularly relevant for CAD patients presenting without standard modifiable CAD risk factors after validation of its predictive capability. If a patient is deemed to be “low risk” according to the prediction model, then a non-invasive diagnostic modality might be preferred in the diagnostic algorithm. Finally, our analysis did not warrant any imputation, sample removal, or variable discount, based on the strength of the ML model to incorporate a large number of variables, including highly correlated ones. Finally, our study could collaborate well with upcoming studies in the fields of prevention and diagnosis of CAD offering a good starting point for addressing the complexity of interrelated metabolites and elucidating potential therapeutic targets.

## 5. Conclusions

In this study, we developed an ML model, utilizing readily available clinical and demographic characteristics combined with a panel of metabolites acquired by a targeted metabolomics approach to predict patients likely to have obstructive CAD on ICA. Implementing ML frameworks of metabolite datasets might further improve clinical decision making in low-to-intermediate risk patients regarding the need for further testing, as well as for the need for preventive therapies. These methods will ultimately contribute to extracting the full potential from metabolomics: to guide clinical decisions and deepen our knowledge of CAD metabolism.

## Figures and Tables

**Table 1:** Baseline clinical and demographic characteristics of the CorLipid trial.

For 958 CORLIPID Patients		N	N %
**Sex**	Female	255	26.6%
	Male	703	73.4%
**Hypertension**	No	398	41.5%
	Yes	560	58.5%
**Diabetes mellitus**	No	642	67.0%
	Yes	316	33.0%
**Dyslipidaemia**	No	594	62.0%
	Yes	363	37.9%
**Family history**	No	788	82.3%
	Yes	169	17.6%
**Smoking**	No	535	55.8%
	Yes	423	44.2%
**Statin administration**	No	487	50.8%
	Yes	455	47.5%
**Age group**	65<	504	52.6%
	65>	452	47.2%
**Previous stroke**	No	929	97.0%
	Yes	28	2.9%
**Peripheral vascular disease**	No	914	95.4%
	Yes	43	4.5%
**Aortic aneurysms**	No	928	96.9%
	Yes	29	3.0%
**Chronic pulmonary obstructive disease**	No	904	94.4%
	Yes	54	5.6%
**Autoimmune disease**	No	941	98.2%
	Yes	17	1.8%
**Atrial fibrillation**	No	858	89.6%
	Yes	100	10.4%
**ACS**	No	425	44.4%
	Yes	533	55.6%
**CAD groups**	NSTEMI	170	17.7%
	STEMI	222	23.2%
	Unstable angina	141	14.7%
	Stable angina	425	44.4%
**Syntax score groups**	0	277	28.9%
	1–22	471	49.2%
	<22	210	21.9%

Data discrepancies are due to missing medical information.

**Table 2:** Baseline continuous clinical characteristics of the CorLipid trial.

	Median	↓95.0% CIs	↑95.0% CIs
**Age**	65	65	66
**Syntax score**	10.0	9.0	12.0
**Body mass index**	28.00	27.80	28.40
**Total cholesterol**	159.0	156.0	163.0
**Triglycerides**	125	122	130
**High-density lipoprotein**	40	39	41
**Low-density lipoprotein**	88	85	92
**High-sensitivity troponin T**	35.0	30.0	46.0
**Low ventricular ejection fraction (%)**	55	55	60

**Table 3:** Descriptive analyses of categorical variables per CAD subgroup.

		CAD Groups								
		**NSTEMI(α)**		**STEMI(β)**		**Unstable Angina(γ)**		**Stable** **Angina(δ)**		(Pair) *p*-Value *
		**N**	**%**	**N**	**%**	**N**	**%**	**N**	**%**	
**Sex**	Female	41	24.10	45	20.30	43	30.50	126	29.60	0.063
	Male	129	75.90	177	79.70	98	69.50	299	70.40	
	Total	170	100.00	222	100.00	141	100.00	425	100.00	
**Hypertension**	No	63	37.10	129	58.10	57	40.40	149	35.10	0.005 (β-α), <0.001 (β-γ), <0.001 (β-δ),
	Yes	107	62.90	93	41.90	84	59.60	276	64.90	
	Total	170	100.00	222	100.00	141	100.00	425	100.00	
**Diabetes mellitus**	No	111	65.30	160	72.10	86	61.00	285	67.10	0.164
	Yes	59	34.70	62	27.90	55	39.00	140	32.90	
	Total	170	100.00	222	100.00	141	100.00	425	100.00	
**Dyslipidemia**	No	104	61.20	166	74.80	92	65.20	232	54.60	0.045 (β-α), <0.001 (β-δ),
	Yes	65	38.20	56	25.20	49	34.80	193	45.40	
	Total	169	100.00	222	100.00	141	100.00	425	100.00	
**Family history**	No	133	78.20	169	76.10	121	85.80	365	85.90	0.012(δ-β)
	Yes	37	21.80	53	23.90	19	13.50	60	14.10	
	Total	170	100.00	222	100.00	140	100.00	425	100.00	
**Smoking**	No	78	45.90	94	42.30	77	54.60	286	67.30	<0.001(δ-α), <0.001(δ-β)
	Yes	92	54.10	128	57.70	64	45.40	139	32.70	
	Total	170	100.00	222	100.00	141	100.00	425	100.00	
**Age (groups)**	65<	93	54.70	143	64.40	67	47.50	201	47.30	0.013 (β-γ), <0.001 (β-δ),
	65>	76	44.70	79	35.60	73	51.80	224	52.70	
	Total	169	100.00	222	100.00	140	100.00	425	100.00	
**Previous stroke**	No	166	97.60	214	96.40	138	97.90	411	96.70	0.602
	Yes	4	2.40	8	3.60	2	1.40	14	3.30	
	Total	170	100.00	222	100.00	140	100.00	425	100.00	
**Peripheral vascular disease**	No	160	94.10	215	96.80	133	94.30	406	95.50	0.53
	Yes	10	5.90	7	3.20	8	5.70	18	4.20	
	Total	170	100.00	222	100.00	141	100.00	424	100.00	
**Aortic aneurysms**	No	167	98.20	220	99.10	141	100.00	400	94.10	0.003 (γ-δ), 0.003 (β-δ), 0.016 (α-δ)
	Yes	2	1.20	2	0.90	0	0.00	25	5.90	
	Total	169	100.00	222	100.00	141	100.00	425	100.00	
**Chronic pulmonary obstructive disease**	No	158	92.90	213	95.90	134	95.00	399	93.90	0.574
	Yes	12	7.10	9	4.10	7	5.00	26	6.10	
	Total	170	100.00	222	100.00	141	100.00	425	100.00	
**Autoimmune disease**	No	167	98.20	219	98.60	137	97.20	418	98.40	0.758
	Yes	3	1.80	3	1.40	4	2.80	7	1.60	
	Total	170	100.00	222	100.00	141	100.00	425	100.00	
**Atrial fibrillation**	No	155	91.20	208	93.70	127	90.10	368	86.60	0.03 (δ-β)
	Yes	15	8.80	14	6.30	14	9.90	57	13.40	
	Total	170	100.00	222	100.00	141	100.00	425	100.00	
**Known CAD**	No	138	81.20	201	90.50	121	85.80	321	75.50	0.318
	Yes	9	5.30	8	3.60	6	4.30	26	6.10	
	Total	147	100.00	209	100.00	127	100.00	347	100.00	
**eGFR < 60**	No	132	77.60	191	86.00	121	85.80	374	88.00	<0.001 (δ-α)
	Yes	38	22.40	29	13.10	19	13.50	41	9.60	
	Total	170	100.00	220	100.00	140	100.00	415	100.00	

* Bonferroni corrected for multiple comparisons Kruskal–Wallis test.

**Table 4:** Descriptive analyses of continuous variables per CAD subgroup.

	CAD Groups								
	**NSTEMI(α)**		**STEMI(β)**		**Unstable** **Angina(γ)**		**Stable** **Angina(δ)**		***p*** -Value * (Pair)
	**Mean**	**±SD**	**Mean**	**±SD**	**Mean**	**±SD**	**Mean**	**±SD**	
**BMI**	27.84	4.33	28.74	4.64	28.35	4.87	28.73	4.54	0.189
**Grace Score**	123	41	125	37	96	32	89	25	<0.001 (δ-α), <0.001 (δ-β), <0.001 (γ-α), <0.001 (γ-β),
**eGFR**	88.2	40.17	98.1	38.42	92.93	33.41	93.17	32.66	0.086
**Total glucose**	122.15	59	134.72	67.83	117.38	42.05	115.37	57.85	0.002 (δ-β), 0.032 (α-β)
**Creatinine**	1.3	1.38	1.04	0.6	1.01	0.79	1.02	0.87	0.076
**Cholesterol**	162.9	46.1	168.9	45.3	162.1	39.1	163.1	41.8	0.648
**Triglycerides**	158	128	158	190	147	72	144	119	0.159
**High-density lipoprotein**	40	13	39	10	42	12	45	14	<0.001 (β-δ), <0.001 (α-δ)
**Low-density lipoprotein**	92	39	101	39	91	34	90	35	0.024 (γ-β)
**High-sensitivity troponin T**	564.5	936	2442.30	2675.80	106.1	397.6	38.5	159.9	<0.001 (δ-α), <0.001 (δ-β), <0.001 (δ-γ,) <0.001 (γ-α), <0.001 (γ-β), <0.001 (α-β),
**Serum glutamic-oxaloacetic transaminase**	42.2	60.8	172	508.2	24	16.9	22.1	17.3	<0.001 (δ-α), <0.001 (δ-β), <0.001 (γ-α), <0.001 (γ-β), <0.001 (α-β),
**Serum glutamic pyruvic transaminase**	297.5	3372.40	78.9	341.8	26.7	25.3	24.3	33.3	0.017 (δ-α), <0.001 (δ-β), <0.001 (γ-β), <0.001 (α-β),
**Lactate dehydrogenase**	308	165	629	601	211	66	222	120	<0.001 (δ-α), <0.001 (δ-β), <0.001 (γ-α), <0.001 (γ-β), <0.001 (α-β),
**Creatine phosphokinase**	317	693	1166	1763	113	159	114	131	<0.001 (δ-α), <0.001 (δ-β), <0.001 (γ-α), <0.001 (γ-β), <0.001 (α-β),
**Low ventricular ejection fraction (%)**	0.5	0.11	0.44	0.1	0.54	0.1	0.56	0.09	<0.001 (δ-α), <0.001 (δ-β), 0.015 (γ-α), <0.001 (γ-β), <0.001 (α-β),

* Bonferroni corrected for multiple comparisons Kruskal–Wallis test.

## Data Availability

Code used in this project is available at the following repository: https://github.com/TheoLiapikos/Syntax_Score_prediction_model_for_CV_patients_using_XGBoost_Classifier (accessed on 27 July 2022).

## Supplementary Materials

The following supporting information can be downloaded at: https://www.mdpi.com/article/10.3390/metabo12090816/s1, Evaluation metrics Equations (S1)–(S10); Table S1: Hyperparameters optimized for extreme gradient boosting classifier (XGBClassifier) predictive algorithm and the ranges of investigated values. The names of the parameters are identical to the names that appear in the corresponding Python library; Table S2. SYNTAX score groups descriptive statistics. Kruskal–Wallis test; Table S3. Biochemical parameters per SYNTAX score group; Table S4. CAD groups with proteins, ceramide, acylcarnitine, and lipid levels; Table S5. Serum levels of proteins, ceramides, and acyl-carnitines by CAD severity.
