# Supplementary material for: Machine Learning Algorithm to Predict Obstructive Coronary Artery Disease: Insights from the CorLipid Trial
Source: Metabolites. 2022 Aug 30;12(9):816. doi: 10.3390/metabo12090816 (PMC9504538; doi:10.3390/metabo12090816)
Supplement: Supplementary file 1 [file metabolites-12-00816-s001.zip › metabolites-1863614-supplementary.pdf]

Supplementary material

Evaluation metrics

1. $TP = \sum_{i=1}^{N} \left(\hat{y}_i = 1 \mid y_i = 1 \right)$
2. $FP = \sum_{i=1}^{N} \left(\hat{y}_i = 1 \mid y_i = 0 \right)$
3. $TN = N_n - FP$
4. $FN = N_p - TP$
5. $Logloss = -\frac{1}{N} \sum_{i=1}^{N} \left[y_i \ln p_i + \left(1 - y_i\right) \ln\left(1 - p_i\right) \right]$
6. $Accuracy = \frac{TP + TN}{TP + TN + FP + FN}$
7. $Precision = \frac{TP}{TP + FP}$
8. $Recall \,/\, Sensitivity = \frac{TP}{TP + FN}$
9. $F1 = 2 * \frac{Precision * Recall}{Precision + Recall}$
10. $Specificity = \frac{TN}{TN + FP}$

Abbreviations:
y : the actual/true value,
$\hat{y}$: the predicted value,
p : the predicted probability for a sample to belong in positive class,
$\ln$: the natural logarithm,
N : the number of samples (observations) in dataset,
N_p : the number of samples in dataset belonging in positive class ($y = 1$),
N_n : the number of samples in dataset belonging in negative class ($y = 0$),

Table S1. Hyperparameters optimized for Extreme Gradient Boosting Classifier (XGBClassifier) predictive algorithm and the ranges of investigated values. The names of the parameters are identical to the names that appear in the corresponding Python library.

Predictive Algorithm	Hyperparameter	Investigated values
XGBClassifier	n_estimators	randint(10, 200)
Extreme Gradient Boosting Classifier	max_depth	randint(1, 12)
	learning_rate	uniform(0.01, 0.25)
	gamma	uniform(0.0, 10.0)
	reg_alpha	uniform(0.0, 10.0)
	reg_lambda	uniform(0.0, 10.0)

uniform() - randomly and uniformly select a float number from the range defined in parentheses; *randint()* - randomly and uniformly select an integer number from the range defined in parentheses

Table S2. SYNTAX Score Groups descriptive statistics.

		Syntax Score Groups						
		**0 (α)**		**1 to 22 (β)**		**>22 (γ)**		**P value*(pair)**
**Sex**	Female	103	37.20%	99	21.00%	53	25.20%	0.009 (α-γ)
	Male	174	62.80%	372	79.00%	157	74.80%	<0.001 (α-β)
	Total	277	100.00%	471	100.00%	210	100.00%	
**Hypertension**	No	132	47.70%	194	41.20%	72	34.30%	0.009 (α-γ)
	Yes	145	52.30%	277	58.80%	138	65.70%	
	Total	277	100.00%	471	100.00%	210	100.00%	
**Diabetes Mellitus**	No	206	74.40%	322	68.40%	114	54.30%	<0.001 (α-γ)
	Yes	71	25.60%	149	31.60%	96	45.70%	0.001 (β-γ)
	Total	277	100.00%	471	100.00%	210	100.00%	
**Dyslipidaemia**	No	180	65.00%	283	60.10%	131	62.40%	0.429
	Yes	97	35.00%	187	39.70%	79	37.60%	
	Total	277	100.00%	470	100.00%	210	100.00%	
**Family history**	No	235	84.80%	383	81.30%	170	81.00%	0.428
	Yes	42	15.20%	87	18.50%	40	19.00%	
	Total	277	100.00%	470	100.00%	210	100.00%	
**Smoking**	No	182	65.70%	231	49.00%	122	58.10%	<0.001 (α-β)
	Yes	95	34.30%	240	51.00%	88	41.90%	
	Total	277	100.00%	471	100.00%	210	100.00%	
**Age (groups)**	65<	150	54.20%	268	56.90%	86	41.00%	<0.001 (β-γ)
	65>	127	45.80%	201	42.70%	124	59.00%	0.01 (α-γ)
	Total	277	100.00%	469	100.00%	210	100.00%	
**Previous Stroke**	No	271	97.80%	456	96.80%	202	96.20%	0.669
	Yes	6	2.20%	15	3.20%	7	3.30%	
	Total	277	100.00%	471	100.00%	209	100.00%	
**Peripheral vascular disease**	No	269	97.10%	452	96.00%	193	91.90%	0.01 (α-γ)
	Yes	7	2.50%	19	4.00%	17	8.10%	
	Total	276	100.00%	471	100.00%	210	100.00%	
**Aortic aneurysms**	No	258	93.10%	466	98.90%	204	97.10%	<0.001 (α-β)
	Yes	19	6.90%	5	1.10%	5	2.40%	0.013 (α-γ)

	Total	277	100.00%	471	100.00%	209	100.00%	
**Chronic pulmonary obstructive disease**	No	258	93.10%	445	94.50%	201	95.70%	0.470
	Yes	19	6.90%	26	5.50%	9	4.30%	
	Total	277	100.00%	471	100.00%	210	100.00%	
**Autoimmune disease**	No	270	97.50%	467	99.20%	204	97.10%	0.099
	Yes	7	2.50%	4	0.80%	6	2.90%	
	Total	277	100.00%	471	100.00%	210	100.00%	
**Atrial fibrillation**	No	235	84.80%	430	91.30%	193	91.90%	0.033 (α-γ)
	Yes	42	15.20%	41	8.70%	17	8.10%	0.015 (α-β)
	Total	277	100.00%	471	100.00%	210	100.00%	
**Known CAD**	No	227	81.90%	384	81.50%	170	81.00%	0.01 (α-γ)
	Yes	5	1.80%	24	5.10%	20	9.50%	
	Total	232	100.00%	408	100.00%	190	100.00%	
**Kidney GFR<55**	No	246	88.80%	412	87.50%	160	76.20%	<0.001 (α-γ)
	Yes	25	9.00%	58	12.30%	44	21.00%	0.005 (β-γ)
	Total	271	100.00%	470	100.00%	204	100.00%	

*** Bonferroni corrected for multiple comparisons Kruskal Wallis test**

Table S3. Biochemical parameters per SYNTAX Score group

	SYNTAX Score						
	0 (α)		1 to 22 (β)		>22 (γ)		
	**Mean(±SD)**	**±SD**	**Mean**	**±SD**	**Mean**	**±SD**	**P value*(pair)**
**BMI**	28.47 (4.8)	4.8	28.61	4.62	28.4	4.23	0.853
**Grace score**	89(31)	31	107	36	119	36	<0.001 (α-β), <0.001 (α-γ), <0.001 (β-γ)
**Total glucose**	96.09(33.16)	33.16	95.65	36.17	84.61	36.59	0.002 (α-γ),0.001 (β-γ)
**Creatinine**	107.86	61.54	123.06	56.66	135.09	57.46	<0.001 (α-β), <0.001 (α-γ), 0.001 (β-γ)
**Cholesterol**	1.01	0.91	1.04	0.74	1.24	1.26	0.006 (α-β), <0.001 (α-γ)
**Triglycerides**	164.5	37.2	164.4	42.8	163.5	50.4	0.445
**High density lipoprotein**	129	117	160	142	156	141	<0.001 (α-β), 0.001 (α-γ)
**Low density lipoprotein**	47	14	41	12	41	12	<0.001 (α-β), <0.001 (α-γ)
**High sensitivity Troponin T**	93	31	94	37	92	43	0.33
**Serum Glutamic-Oxaloacetic Transaminase**	113.8	541.4	983.7	1967.2	862.1	1760	<0.001 (α-β), <0.001 (α-γ)
**Serum Glutamic Pyruvic Transaminase**	27.3	41.4	81.1	352.6	60.5	109	<0.001 (α-β), <0.001 (α-γ)
**Lactate Dehydrogenase**	191.1	2672.1	48	236	36.9	81.5	0.001 (α-β)
**Creatine Phosphokinase**	227	77	366	404	399	424	<0.001 (α-β), <0.001 (α-γ)
**Low ventricular ejection fraction (%)**	145	415	523	1227	472	986	<0.001 (α-β), <0.001 (α-γ)
**Total glucose**	0.54	0.1	0.5	0.11	0.48	0.12	<0.001 (α-β), <0.001 (α-γ)

* Bonferroni corrected for multiple comparisons Kruskal Wallis test

Table S4. CAD groups with proteins, ceramide, acylcarnitine and lipid levels

	CAD Groups																
	NSTEMI				STEMI				Unstable Angina				Stable Angina				P value* (pair)
	N	Median	↓95.0% CIs	↑95.0% CIs	N	Median	↓95.0% CIs	↑95.0% CIs	N	Median	↓95.0% CIs	↑95.0% CIs	N	Median	↓95.0% CIs	↑95.0% CIs	
**Proteins (N)**																	
**Galectin (ng/ml) (N=932)**	164	8.41	7.6	9.4	216	10	9.1	10.9	136	9.36	8.87	10.9	416	9.82	9.3	10.4	0.099
**NGAL(ng/ml)(119)**	32	4.6	3	6.5	26	3.95	2	6.5	17	1.3	0.9	2.5	44	1.8	1.3	4.5	0.009 (δ-γ) 0.002 (γ-α)
**Adiponectin (ng/ml)(N=216)**	35	162	151.7	170	41	164	157	172	38	162.5	159	175	102	161	156	168	0.957
**ApoB/ApoA-I(405)**	66	0.86	0.77	1.15	112	0.93	0.85	1.01	64	0.82	0.66	0.97	163	0.75	0.68	0.86	0.11
**Ceramides(N=915)**																	
**C16:0**	163	0.546	0.513	0.571	214	0.629	0.574	0.654	134	0.52	0.48	0.54	404	0.487	0.471	0.511	0.01 (δ-α), <0.001 (δ-β),<0.001 (γ-α)
**C18:0**		0.214	0.196	0.237		0.243	0.225	0.272		0.192	0.186	0.216		0.176	0.164	0.19	<0.001 (δ-α), <0.001 (δ-β), 0.001 (γ-α)
**C24:0**		6.802	6.144	7.922		7.571	7.042	8.392		6.73	6.033	7.426		6.845	6.52	7.324	0.020 (γ-β), 0.029 (δ-β)
**C24:1**		3.137	2.889	3.66		3.566	3.317	3.866		2.859	2.614	3.3		2.962	2.872	3.146	0.001 (γ-β),<0.001 (δ-β)
**Ratio C16:0/C24:0**		0.081	0.076	0.087		0.073	0.07	0.081		0.073	0.069	0.078		0.07	0.067	0.074	0.002 (δ-α)
**Ratio C18:0/C24:0**		0.031	0.028	0.035		0.033	0.031	0.035		0.031	0.026	0.034		0.025	0.024	0.026	<0.001 (δ-α), <0.001 (δ-β), 0.01 (δ-γ)
**Ratio C24:1/C24:0**		0.457	0.434	0.511		0.456	0.428	0.48		0.454	0.425	0.49		0.436	0.419	0.459	0.329
**Acyl L-carnitines (N=946)**																	

**C2**	170	2906.5	2649.44	3154.25	219	2770.56	2539.3	3045.37	140	3147.27	2839.91	3452.4	417	2969.54	2846.84	3111.7	0.219
**C3**		168.35	151.99	182.39	219	173.55	157.15	191.74	140	181.15	167.93	196.96		176.8	169.84	185.38	0.824
**C4**		39.92	36.2	44.81		38.1	35.6	42.41		37.99	34.83	43.02		38.62	36.74	40.81	0.782
**C5**		24.79	23.46	28.8		29.08	26.36	30.73		25.13	22.86	27.7		25.72	24.95	27.5	0.026 (γ-β)
**C6**		27.46	25.83	31		27.58	25.65	29.1		30.69	27.77	33.83		29.98	28.87	31.46	0.329
**C8**		54.66	49	60.09		52.99	49.11	58.13		56.6	51.9	66.67		63.06	58.68	68.55	0.053
**C10**		89.88	78.25	105.42		86.53	79.36	94.45		91.5	83.49	110.42		106.09	96.74	116.37	0.019 (δ-β)
**C12**		26.54	23.7	30.2		27.96	25.11	29.83		29.17	24.86	31.54		29.88	28.26	31.39	0.377
**C14**		17.51	16.27	19.55		17.78	17.04	18.86		19.28	17.52	20.69		19.17	18.43	19.79	0.301
**C16**		58.38	55.03	63.64		58.29	55.82	60.89		62.9	60.82	66.52		63.21	60.85	65.63	0.012 (δ-β)
**C18**		17.35	16.09	18.35		18.63	17.76	19.54		18.63	17.49	19.29		19.01	18.45	19.59	0.137
**C18:1**		85.82	78.64	94.91		82.8	76.43	88.61		91.3	79.31	97.06		92.53	88.15	97.76	0.013 (δ-β)
**C18:2**		54.86	50.74	59.54		50.26	47.34	52.41		53.75	50	60.48		60.21	57.89	63.29	<0.001 (δ-β)

Fatty Acids (N=462)

**C10:0**	91	14.67	13.87	16.13	92	15.98	15.64	16.94	95	16.41	15.29	17.13	184	13.18	12.11	14.02	<0.001 (δ-β), <0.001 (γ-δ)
**C12:0**		28.73	27.81	31.14		29.44	28.54	30.65		29.62	28.43	31.41		23.16	20.82	26.11	0.003 (δ-β), <0.001 (γ-δ)
**C14:0**		49.89	41.67	59.51		47.99	42.57	60.06		63.19	55.12	71.9		50.39	43.27	58.8	0.064
**C14:1**		42.04	40.56	46.1		44.35	41.28	49.25		42.63	40.58	49.2		33.71	32.62	35.9	0.023 (δ-β), 0.042 (γ-δ)
**C15:0**		16.28	14.15	17.78		15.66	14.29	17.12		17.39	15.72	19.14		14.99	14.01	15.87	0.005 (δ-γ)
**C16:0**		1783.42	1516.81	2180.78		1806.5	1600.63	1949.54		1917.14	1644.89	2214.48		1270.19	1058.03	1424.22	<0.001 (δ-α), <0.001 (δ-β), <0.001 (δ-γ)
**C16:1**		94.15	79.93	118.22		99.42	83.32	113.49		106.56	92.24	123.12		68.97	57.24	80.95	<0.001 (δ-α), <0.001 (δ-β), <0.001 (δ-γ)

**C17:0**	21.01	17.67	24.46	20.52	17.25	22.4	22.7	18.68	24.4	13.13	11.68	15.85	<0.001 (δ-α), <0.001 (δ-β), <0.001 (δ-γ)
**C17:1**	117.66	116.37	119.77	116.88	115.79	118.2	117.93	117.12	122.37	98.18	80.71	112.47	<0.001 (δ-α), <0.001 (δ-β), <0.001 (δ-γ)
**C18:0**	479.75	418.05	581.89	515.56	411.87	602.13	549.42	458.23	632.56	373.24	300.2	431.84	0.003 (δ-α), 0.002 (δ-β), <0.001 (δ-γ)
**cis_C18:1**	1828.52	1687.32	2170.46	1765.11	1571.01	2024.97	1967.38	1721.87	2258.5	1413.07	1286.01	1575.49	<0.001 (δ-α), 0.002 (δ-β), <0.001 (δ-γ)
**cis_C18:2**	1852.94	1633.86	2049.21	1898.33	1699.97	2056.83	1903.79	1696.14	2232.47	1775.62	1600.89	1975.63	0.583
**C18:3 n6**	102.55	98.75	107.07	109.46	101.82	115.19	108.91	103.83	118.88	96.21	93.98	101.7	0.003 (δ-β), <0.001 (δ-γ)
**C18:3 n3**	90.57	85	97	94.93	89.27	99.13	96.43	90.85	99.01	83.57	81.96	84.79	0.004 (δ-α), <0.001 (δ-β), <0.001 (δ-γ)
**C20:1n11**	60.52	56.47	61.49	60.45	59.25	61.62	60.93	59.75	62.37	62.36	61.64	63.3	0.003 (δ-β)
**C21:0**	7.08	6.06	7.6	7.13	6.2	7.66	7.34	6.98	7.64	7	6.94	7.05	0.185
**C20:2 cis**	44.22	41.78	46.71	42.41	40.59	43.22	44.36	43.11	46.26	63.81	47.96	65.68	<0.001 (δ-β)
**C22:0**	73.71	63.53	74.97	74.51	73.15	75.63	74.07	72.4	75.38	48.56	47.58	51.34	<0.001 (δ-α), <0.001 (δ-β), <0.001 (δ-γ)

**C20:3 w6**		101.15	90.57	114.25		104.12	92.04	114.01		105.53	95.31	115.33		83.78	75.55	92.55	0.007 (δ-α), 0.005 (δ-β), 0.003 (δ-γ)
**C20:4 w6**		394.16	367.48	452.8		378.79	323.35	414.4		406.65	379.62	462.35		347.42	321.03	368.23	0.042 (δ-α), 0.012 (δ-γ)
**C23:0**		9.48	7.67	10.13		10.39	9.06	10.83		9.97	8.8	10.79		4.81	4.36	5.73	<0.001 (δ-α), <0.001 (δ-β), <0.001 (δ-γ)
**C20:5 w3 cis**		64.11	62.87	67.2		64.74	63.59	65.49		66.14	64.54	68.77		64.19	62.93	65.58	0.306
**C24:0**		62.57	60.94	67.55		67.07	62.41	68.66		65.08	61.19	68.42		43.39	40.58	46.08	<0.001 (δ-α), <0.001 (δ-β), <0.001 (δ-γ)
**C24:1**	90	67.34	65.38	68.55	88	66.47	64.43	67.51	94	67.34	65.36	69.46	181	63.44	62.49	64.39	<0.001 (δ-α), 0.002 (δ-β), <0.001 (δ-γ)
**C22:6 w3 cis**		95.63	91.47	100.97		100.87	92.5	106.34		102.09	95.85	110.54		93.92	90.43	98.55	0.169

* Bonferroni corrected for multiple comparisons Kruskal Wallis test

Table S5. Serum levels of proteins, ceramides and acyl-carnitines by CAD severity

	Syntax Score Groups												
	0 (α)				1 to 22 (β)				>22 (γ)				P value (pair) *
	N	Median	↓95.0% CIs	↑95.0% CIs	N	Median	↓95.0% CIs	↑95.0% CIs	N	Median	↓95.0% CIs	↑95.0% CIs	
**Proteins (N)**													
**Galectin (ng/ml) (N=932)**	270	10.3	9.3	10.9	457	9.1	8.7	9.77	205	10.1	9.55	11.27	0.065 (β-γ)
**NGAL(ng/ml)(119)**	22	1.75	0.7	5.3	57	2.4	1.8	4.2	40	3.2	1.9	6.5	0.29
**Adiponectin (ng/ml)(N=216)**	48	168	160	173	100	160	156	169	68	161.1	156	167	0.856
**ApoB/ApoA-I(405)**	134	0.69	0.63	0.8	194	0.88	0.82	0.96	77	0.86	0.76	1.05	0.005 (α-β)
**Ceramides (N=915)**													
**C16:0**	269	0.517	0.476	0.533	443	0.526	0.507	0.55	203	0.547	0.528	0.572	0.052 (α-γ)
**C18:0**	269	0.187	0.171	0.208	443	0.202	0.19	0.214	203	0.209	0.197	0.229	0.049 (α-β), 0.032 (α-γ)
**C24:0**	269	6.76	6.324	7.434	443	7.195	6.648	7.443	203	7.272	6.725	7.657	0.592
**C24:1**	269	2.906	2.837	3.197	443	3.18	3.069	3.352	203	3.223	3.06	3.506	0.052 (α-γ)
**Ratio C16:0/C24:0**	269	0.072	0.067	0.075	443	0.073	0.071	0.076	203	0.077	0.071	0.082	0.168
**Ratio C18:0/C24:0**	269	0.026	0.025	0.028	443	0.03	0.028	0.031	203	0.029	0.027	0.032	0.052 (α-β), 0.055 (α-γ)
**Ratio C24:1/C24:0**	269	0.434	0.414	0.452	443	0.459	0.435	0.475	203	0.454	0.43	0.48	0.19
**Acyl L-carnitines (N=946)**													
**C2**	273	3013.09	2848.26	3146.54	466	2841.43	2652.45	3047.79	207	2951.55	2798.57	3325.86	0.132
**C3**	273	171.49	162.86	179.56	466	175.68	167.86	184.94	207	186.56	170.45	209.04	0.084
**C4**	273	36.96	34.21	40.46	466	37.95	35.6	40.18	207	45.16	38.94	49.61	0.002 (α-γ), 0.005 (β-γ)
**C5**	273	25.25	23.99	26.36	466	26.41	24.95	28.63	207	27.82	25.34	30.79	0.024 (α-γ)
**C6**	273	29.81	28.07	33.04	466	28.89	27.63	30.08	207	28.88	27.1	31.34	0.889
**C8**	273	60.09	56.03	66.36	466	57.65	53.41	61.45	207	55.13	49.58	62.7	0.521
**C10**	273	99.63	90.03	109.52	466	94.48	86.47	101.15	207	92.32	80.54	104.81	0.43
**C12**	273	29.55	27.41	31.17	466	28.53	26.63	30.33	207	27.73	25.11	29.71	0.445
**C14**	273	19.24	18.12	20.36	466	18.54	17.59	19.27	207	18.21	17.06	19.69	0.229

**C16**	273	65.18	62.57	67.9	466	60.28	57.95	62.48	207	59.27	56.28	61.94	0.044 (α-β), 0.031(α-γ)
**C18**	273	19.11	18.27	20.08	466	18.39	17.7	19.03	207	18.18	17.5	19.08	0.166
**C18:1**	273	91.7	85.27	98.82	466	88.03	82.8	92.55	207	87.63	80.05	93.49	0.392
**C18:2**	273	60.48	56.37	64.61	466	53.83	51.35	56.62	207	53.28	49.37	57.57	0.019 (α-β), 0.012 (α-γ)
						**Fatty Acids (N=462)**							
**C10:0**	139	14.94	14.12	16.05	225	15.29	14.05	15.86	98	14.29	13.67	16.09	0.846
**C12:0**	139	28.38	26.4	29.56	225	28.7	27.96	29.47	98	26.69	21.75	28.09	0.107
**C14:0**	139	55.96	50.6	62.71	225	53.33	47	61.4	98	46.89	40.88	55.89	0.127
**C14:1**	139	40.84	34.73	43.44	225	41.14	40.35	42.06	98	40.76	31.05	48.63	0.589
**C15:0**	139	16.02	14.85	16.84	225	15.78	14.96	16.67	98	15.2	13.95	17.1	0.943
**C16:0**	139	1544.06	1362.65	1673.26	225	1643.83	1555.12	1828.46	98	1609.08	1372.24	1859.12	0.352
**C16:1**	139	85.08	76.05	105.34	225	92.22	82.63	98.1	98	78.78	73.05	94.68	0.37
**C17:0**	139	17.67	14.4	19.79	225	18.22	16.75	20.42	98	18.14	14.72	20.4	0.543
**C17:1**	139	115.78	114.35	116.85	225	115.56	114.8	116.39	98	117.46	116	118.72	0.036 (α-γ)
**C18:0**	139	446.6	396.67	505.74	225	467.38	412.25	529.82	98	446.83	347.43	522.85	0.859
**cis_C18:1**	139	1550.19	1388.97	1709.86	225	1814.8	1687.32	1893.56	98	1648.71	1470.54	1804.82	0.022 (α-β)
**cis_C18:2**	139	1915.14	1719.39	2020.69	225	1834.87	1678.13	1931.15	98	1859.13	1607.59	2101.85	0.874
**C18:3 n6**	139	103.83	100.34	110.49	225	103.52	100.49	107.56	98	101.51	98.75	106.37	0.636
**C18:3 n3**	139	90.05	84.82	94.49	225	89.27	85.42	91.73	98	89.38	85	96.64	0.64
**C20:1n11**	139	61.57	60.34	62.37	225	61.5	60.75	62.1	98	60.9	59.81	61.78	0.909
**C21:0**	139	7.03	6.97	7.16	225	6.98	6.89	7.08	98	7.23	7.08	7.49	0.116
**C20:2 cis**	139	46.07	43.97	49.28	225	44.36	43.14	46.91	98	45.8	43.24	50.49	0.597
**C22:0**	139	60.37	49.64	73.87	225	68.91	52.81	74.07	98	63.08	54.44	73.9	0.739
**C20:3 w6**	139	95.31	86.93	102.27	225	96.86	87.79	106.25	98	94.81	85.71	102.63	0.708
**C20:4 w6**	139	360.33	336.72	392.46	225	383.75	352.4	406.41	98	386.27	333.97	414.34	0.685
**C23:0**	139	7.85	5.57	9.59	225	8.14	6.49	9.63	98	7.83	5.97	9.86	0.695
**C20:5 w3 cis**	139	65.33	63.98	66.75	225	63.97	63.14	64.98	98	65.53	64.11	68.07	0.108

**C24:0**	139	61.29	46.08	64.93	225	61.54	59.94	62.56	98	59.63	48.34	67.55	0.76
**C24:1**	138	64.22	63.36	65.12	218	65.36	63.87	66.63	97	66.49	64.91	67.62	0.135
**C22:6 w3 cis**	139	96.86	91.97	100.2	225	94.29	91.86	100.69	98	100.57	94.83	105.82	0.282

* Bonferroni corrected for multiple comparisons Kruskal Wallis test
